# Identification of Novel MicroRNAs in Primates by Using the Synteny Information and Small RNA Deep Sequencing Data

**DOI:** 10.3390/ijms141020820

**Published:** 2013-10-16

**Authors:** Zhidong Yuan, Hongde Liu, Yumin Nie, Suping Ding, Mingli Yan, Shuhua Tan, Yuanchang Jin, Xiao Sun

**Affiliations:** 1 School of Life Sciences, Hunan University of Science and Technology, Xiangtan 411201, China; E-Mails: zdyuan@hnust.edu.cn (Z.Y.); ding4130@gmail.com (S.D.); mlyan@hnust.edu.cn (M.Y.); shtan@hnust.edu.cn (S.T.); jinyuanchang2013@gmail.com (Y.J.); 2 State Key Laboratory of Bioelectronics, School of Biological Science and Medical Engineering, Southeast University, Nanjing 210096, China; E-Mails: liuhongde@seu.edu.cn (H.L.); ym_nie@seu.edu.cn (Y.N.)

**Keywords:** genome-wide, miRNA, mammalian genome, synteny, deep sequencing

## Abstract

Current technologies that are used for genome-wide microRNA (miRNA) prediction are mainly based on BLAST tool. They often produce a large number of false positives. Here, we describe an effective approach for identifying orthologous pre-miRNAs in several primates based on syntenic information. Some of them have been validated by small RNA high throughput sequencing data. This approach uses the synteny information and experimentally validated miRNAs of human, and incorporates currently available algorithms and tools to identify the pre-miRNAs in five other primates. First, we identified 929 potential pre-miRNAs in the marmoset in which miRNAs have not yet been reported. Then, we predicted the miRNAs in other primates, and we successfully re-identified most of the published miRNAs and found 721, 979, 650 and 639 new potential pre-miRNAs in chimpanzee, gorilla, orangutan and rhesus macaque, respectively. Furthermore, the miRNA transcriptome in the four primates have been re-analyzed and some novel predicted miRNAs have been supported by the small RNA sequencing data. Finally, we analyzed the potential functions of those validated miRNAs and explored the regulatory elements and transcription factors of some validated miRNA genes of interest. The results show that our approach can effectively identify novel miRNAs and some miRNAs that supported by small RNA sequencing data maybe play roles in the nervous system.

## 1. Introduction

MicroRNA (miRNA) is a vital class of regulated RNA. miRNAs regulate gene expression by base pairing to target mRNAs causing translation repression or mRNA degradation. miRNAs were identified usually by molecular cloning and sequencing technologies. These methods tend to be highly biased towards abundantly and/or ubiquitously expressed miRNAs [1–3] and, in addition, certain miRNAs are likely to be missed during sequence cloning procedures [3].

A number of computational methods to identify miRNAs in an organism have been published. Most of these methods use the BLAST program [4] to generate local sequence alignments that are used to identify homologous pre-miRNAs [5–7]; a few of these methods use the BLAT tool [8] to search for candidate pre-miRNAs [9,10]. One of the disadvantages of using BLAST is that the region that is aligned corresponds to an optimal local alignment. miRNAs from the same family often diverge at the 5′ and 3′ ends of the genes in distantly-related organisms. In such cases, the optimal local BLAST alignments do not extend enough to cover the entire length of the pre-miRNA. In a number of studies, when the short mature miRNAs sequences were used as query sequences in BLAST searches, two putative miRNA hairpin structural regions were extracted by extending the aligned sequence upstream and downstream of the BLAST alignments [6,11]. Although the BLAST method has high sensitivity, it may produce a high number of false positives because no potential transcriptional information which could be predicted as pre-miRNAs is available.

The rapid development of sequencing technology has led to more and more genomic sequences becoming available and web sites such as the UCSC Genome Browse [12] have published global alignments of the genomic data of two or more organisms [13,14]. Because most miRNAs are conserved, especially in two closely-related organisms, it should be possible to obtain the syntenic homologous miRNAs of an organism using comparative genomics if the miRNAs of another closely-related organism are known. Whole genome alignments should make it possible to use syntenic homologous blocks to predict pre-miRNAs, and some of the problems of using the prediction methods based on BLAST will be avoided, making the identification of pre-miRNAs more reliable.

More recently, the newly developed deep sequencing technologies have been used to detect small RNAs [15–20]. Deep sequencing is a very effective method for the large-scale discovery of small non-coding RNAs. Especially, this approach could identify poorly expressed or species-specific miRNAs [21]. However, in many cases, deep sequencing is used to uncover miRNAs that are expressed in limited conditions. Some miRNAs are expressed in response to a particular external environmental stimulation, some are expressed at a specific developmental or disease stage, and others are expressed only in some tissues. Fortunately, we could use comparative genomics method to predict their homologs in our organism of interest based on some poorly expressed or species-specific miRNAs that have been identified in other organisms. In contrast with human, far less miRNAs in other primates have been documented. To uncover novel miRNAs in some primates, we discovered novel miRNAs by an effective computational approach based on syntenic information. Meanwhile, we re-analyzed small RNA sequencing data in four primates and the results also supported some computational results. Moreover, we analyzed the potential functions of those validated miRNAs and we also explored their transcription factor and corresponding binding sites among the potential regulatory sequences of some validated miRNA genes of interest.

## 2. Results and Discussion

### 2.1. Computational Identification of Marmoset Pre-miRNAs

Pre-miRNAs are usually located in highly conserved syntenic regions in closely related species. It enables identification of miRNAs based on synteny information among closely related species. To test the effectiveness of our method, we first used it to identify candidate pre-miRNAs in the marmoset genome with the well-studied human miRNA data (Table 1). Marmosets are arboreal primates and the smallest monkey in the world. As yet, no studies on marmoset miRNAs have been published. Fortunately, we identified 1288 candidate miRNA loci and sequences in marmoset using synteny information between marmoset and human genome. From these candidates, 929 of them were determined as potential pre-miRNAs by MiPred (Southeast University, Nanjing, China) [22] (Table 1; the sequences and genome coordinates are listed in Supplementary Information 1 [23] and Table S1). Further analysis revealed that only 387 pre-miRNAs could be classified into 225 known miRNA families according to the miRNA family information in the current miRBase (The University of Manchester, Manchester, UK) [24] (Table S2). The miR-515 family with 28 pre-miRNA members is the biggest miRNA family that was annotated by the Infernal tool in the marmoset genome (Table S2). In the human genome, the largest miRNA family in the current miRBase is the miR-548 family with 69 pre-miRNA members; the second largest is the miR-515 family with 42 members. The Infernal tool lacks sufficient power to classify miR-548 family; only 12 members of the miR-548 family have been identified in humans, and none of the 31 miR-548 precursors have been identified in marmoset, although they are homologs of human pre-miRNAs. The sequences of these members show low complexity and they are derived from MADE1 element [25,26].

### 2.2. Computational Identification of Pre-miRNAs in the Other Four Primates

We also used the computational approach to predict the pre-miRNAs in four other primates: chimpanzee, gorilla, orangutan and rhesus macaque, which share a close genetic relationship with human and marmoset (Table 1). The already known miRNAs of the four species are documented in miRBase 19. We identified 1324 candidate pre-miRNAs in the chimpanzee genome that are homologs of the human pre-miRNAs and 601 of the 655 chimpanzee pre-miRNAs documented in miRBase 19 were re-identified in our study. Thus, our method successfully re-identified 91.76% of the known pre-miRNAs and a further 721 new chimpanzee pre-miRNAs were identified (Table 1, Supplementary Information 1 and Table S1). In the orangutan genome, 590 of the 633 (93.21%) known pre-miRNAs documented in miRBase 19 were re-identified and 650 new orangutan pre-miRNAs were identified (Table 1, Supplementary Information 1 and Table S1). Moreover, in gorilla and rhesus macaque, 301 of 322 (93.48%) and 480 of 535 (89.72%) known pre-miRNAs were re-identified respectively. In total, 979 new gollira pre-miRNAs and 639 new pre-miRNAs in the rhesus macque were discovered in this study (Table 1, Supplementary Information 1 and Table S1).

Among the newly found pre-miRNAs, only 46 pre-miRNAs of the chimpanzee have been annotated into 40 known miRNA families (Table S2), 197 pre-miRNAs of gorilla have been classified into 127 known miRNA families (Table S2), and 50 pre-miRNAs have been annotated into 38 known miRNA families in the orangutan while 50 pre-miRNAs have been classified into 34 known miRNA families in rhesus macaque (Table S2) by using the Infernal tool [27]. After analyzing those newly identified pre-miRNAs, we also found there are 172 conservative pre-miRNAs across human, chimpanzee, gorilla, orangutan, rhesus macaque and marmoset (Table S3).

### 2.3. Novel Predicted Pre-miRNAs Validated by High Throughput Small RNA Sequencing Data

**Figure 1:**
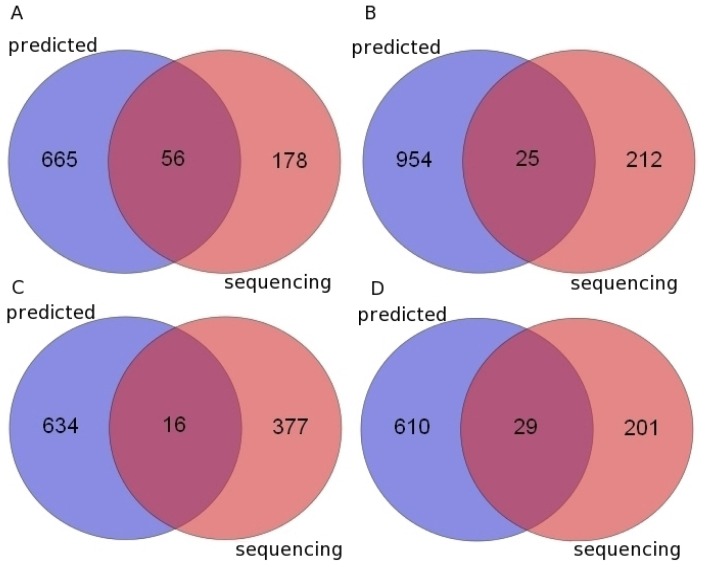
The Venn diagrams of miRNAs identified by using a comparative genomics approach and small RNA sequencing data. (**A**) Chimpanzee; (**B**) Gorilla; (**C**) Orangutan; (**D**) Rhesus macaque. Predicted: miRNAs which were predicted by using a comparative genomics approach. Sequencing: miRNAs which were identified from small RNA sequencing data.

Here, the small RNA deep sequencing data is derived from the brain samples in chimpanzee and rhesus macaque and the brain and liver samples in gorilla and orangutan [28,29]. These data have been re-analyzed to discover novel miRNAs to support our previous computational results. After processing with miRDeep2 (The Max Delbrück Center for Molecular Medicine, Berlin, Germany) [30] and filtering with a positive score and a significant *p*-value, we found 234 new pre-miRNAs (66 with expression evidence for star sequence) in the chimpanzee, 237 (53 with expression evidence for star sequence) in the gorilla, 393 (67 with expression evidence for star sequence) in the orangutan and 230 (62 with expression evidence for star sequence) in the rhesus macaque (Tables 1, S4, Figure 1).

We found 56, 25, 16 and 29 predicted pre-miRNAs have been validated by the small RNA sequencing data in chimpanzee, gorilla, orangutan and rhesus macaque, respectively (Figure 1, Tables 1, S4 and S5). Those were far less than our previous predicted results (Figure 1, Table S1). The main reason is that miRNAs expression response to environmental stimulus in different tissues at different development stages or different pathological states, and the miRNAs were detected only from several tissues could not represent all the miRNAs in the whole body. In this work, the novel miRNAs and pre-miRNAs were identified in the miRNA transcriptom data (Table S4) which is only originated from the tissues of brain or liver [28,29], while the pre-miRNAs (Table S1) were predicted by comparative genomics method may express in various tissues at different developmental stages due to the origin of their homologs in human are diversity. Furthermore, the available miRNA transcriptome data of the four primates is also very limited.

### 2.4. The Interesting Functional and Biological Importance of These Validated miRNAs

As the small RNA deep sequencing data is derived from the brain samples in chimpanzee and rhesus macaque and the brain and liver samples in gorilla and orangutan, some validated miRNAs (Figure 1, Tables S4 and S5) maybe play roles in the nervous system. To explore the biological processes that the validated mRNAs in these four primates are involved, we obtained their predicted targets using TargetScan (Whitehead Institute for Biomedical Research, Cambridge, MA, USA) [31] and PITA (Weizmann Institute, Rehovot, Israel) [32]. Moreover, 311, 471, 420 and 69 common targets of these miRNAs were identified in chimpanzee, gorilla, orangutan and rhesus macaque, respectively. After doing functional enrichment analyses of these common targets in each organism, we found especially the targets of 13 validated miRNAs (Table S6) in orangutan showed significant over-representation of 12 Gene ontology (GO) terms involved in nervous system, such as “neuron apoptotic process”, “regulation of neuron apoptotic process”, “negative regulation of neuron apoptotic process” and so on (Table S7). Moreover, in chimpanzee, those targets significantly involved in two GO terms involved in nervous system and they are “ciliary neurotrophic factor-mediated signaling pathway” and “glial cell differentiation” (Table S7) and the corresponding 11 miRNAs are listed in Table S6. However, for those validated miRNAs and their targets of the other two primates, we have not seen obvious signals involved in nervous system (Table S7).

### 2.5. The Potential Transcription Factor Binding Sites of the Validated miRNA Genes Which Involve in Nervous System

Since the target genes of the miRNAs (Table S6) have been inferred to play roles in the brain, could we find some brain-specific regulatory information among those miRNAs? As there lacks enough tissue expression information in chimpanzee and orangutan, it is hard to find their tissue-specific regulatory sequences, but we could maybe find some common transcription factor binding sites (TFBSs) between them. Previous studies showed some intronic miRNAs have independent regulatory elements while other intronic miRNAs share the regulatory elements with their host genes [33]. Moreover, nucleosomes can inhibit or facilitate transcription factor (TF) binding, and TFBSs tend to be located in low nucleosome occupancy or nucleosome-depleted regions [34,35]. Thus, it was feasible to predict the TFs and TFBSs based on the sequences and their nucleosome position information. To do this, we first classified the miRNAs into intronic miRNAs and intergenic miRNAs based on their genomic contexts and we found 12 intronic miRNAs and 12 intergenic miRNAs among the 24 miRNAs (Tables 2, S6). Then, we scanned their potential upstream regulatory sequences of those miRNAs combining with the nucleosome-DNA interaction model [36,37] and vertebrate position frequency matrices (PFMs) from JASPAR (University of Copenhagen, Copenhagen, Denmark) [38]. Moreover, in the chimpanzee, only the common binding sites of Hltf have been found locating in the upstream sequences of two out of three intergenic miRNA genes and no motifs have been found in the upstream sequence of intergenic ptr-mir-2964a (Table S6). ETS1, FOXL1 and Hltf are the three common TFs which binding sites are locating in the upstream sequences of six out of seven miRNA host genes in the chimpanzee (Tables S6, S8). Moreover, the binding sites of 19 TFs are located in the sequences between the start sites of the intron and the start sites of five intronic miRNA genes respectively and the five miRNAs are ptr-mir-2115, ptr-mir-2681, ptr-mir-4511, ptr-mir-4760 and ptr-mir-4762 (Tables S6, S8). However, no TFBSs have been found in the potential regulatory sequences of the other two intronic miRNAs: ptr-mir-548t and ptr-mir-4691 (Tables S6, S8). We inferred that the two intronic miRNA genes may share regulatory elements with their host genes and the 19 TFs of the intronic miRNAs are also including the previous Hltf, ETS1 and FOXL1. While in the orangutan, the binding sites of GATA2 have been discovered in the upstream sequences of the five intergenic miRNA genes except ppy-mir-200b, ppy-mir-203b and ppy-mir-3065 (Table S6). Eight common TFs: ETS1, FOXC1, FOXL1, GATA2, HOXA5, MZF1_1-4, Nkx2-5 and SPIB could bind the upstream sequences of the two out of three intronic miRNAs and no TFBSs have been found in the potential regulatory sequences of ppy-mir-301a and ppy-mir-4419a (Tables S6, S8). Moreover, ETS1, FOXC1, GATA3, Nkx2-5 and SOX10 could bind the upstream sequences of the three out of four intronic miRNAs (Tables S6, S8). In all, although we have determined these potential TFs and TFBSs by computational methods, the results with available experimental evidence are always more convincing.

## 3. Experimental Section

### 3.1. Data sources

The sequences and genome coordinates of the miRNAs used in this study were obtained from miRBase 19 [24]. The LiftOver conversion data for the whole genome alignment of human sequence (reference sequence) with marmoset, chimpanzee, gorilla, orangutan and rhesus macaque were downloaded from UCSC Genome Browser [12]. The LiftOver utility [39] translates genomic coordinates between assemblies.

The deep sequencing data of the chimpanzee and rhesus macaque was downloaded from NCBI GEO database (GSE26545) (National Center for Biotechnology Information, Bethesda, MD, USA) [29], and the small RNA sequencing data of the gorilla and orangutan came from European Bioinformatics Institute (Hinxton, Cambridge, UK) with the study accession number ERP000973 [28].

### 3.2. A Comparative Genomics Method for the Identification of Orthologous Pre-miRNAs

**Figure 2:**
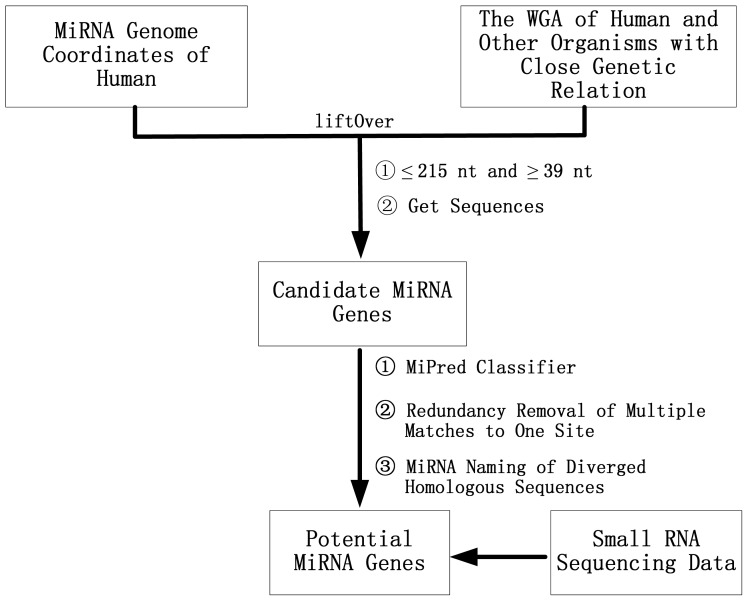
The flow chart used to identify novel miRNAs by using a comparative genomics approach and small RNA sequencing data. WGA: whole genome alignment.

Human was selected as the reference organism because, to date, the human miRNAs are the most studied and have the most experimental evidence, and humans are closely related to other primates such as chimpanzee, gorilla, orangutan, rhesus macaque and marmoset. Using a comparative analysis method, the pre-miRNAs of marmoset, chimpanzee, gorilla, orangutan and rhesus macaqu have been investigated. The comparative genomics method in this study is explained as follows (see flow chart Figure 2): first, the LiftOver program [39] was used to map the genome coordinates of the human pre-miRNAs to the genomes of other closely related species to obtain the coordinates of the candidate homologous miRNAs on the genomes of the interested organisms; next, if different members of one miRNA family mapped to overlapping loci, then only one miRNA was selected to represent this miRNA family in that organism; after retrieving the sequences using the coordinates of the candidate pre-miRNAs they were classified using the MiPred program [22] with the modified threshold value of gene lengths based on the sequence data of animals that are available in miRBase; and the newly identified homologous sequences of the human pre-miRNAs were named according to the miRBase naming criteria [24]. Finally, The potential pre-miRNAs were annotated and classified into the known miRNA families by program rfam_scan.pl, which is a perl wrapper for searching nucleotide sequences against the Rfam database (v11.0) Wellcome Trust Sanger Institute, Hinxton, Cambridge, UK) [40] using the Infernal software (HHMI’s Janelia Farm Research Campus, Ashburn, VA, USA) [27]. Moreover, our scripts for the identification of orthologous pre-miRNAs are available in Supplementary information 2.

To test the effectiveness of the method, it was first applied to identify candidate marmoset miRNAs. Marmoset miRNAs are not available in miRBase and had never been reported yet. Moreover, we used it to identify miRNAs in chimpanzee, gorilla, orangutan and rhesus macaque, while some of their miRNAs have been reported and documented. The overlap ratio between the genome coordinates of computationally identified pre-miRNAs with the documented pre-miRNAs was calculated using the following formula:

(1) Overlap ratio=Number of overlapping bases/The length of the corresponding miRNAs

when the overlap ratio is no less than 0.5, the pre-miRNA which overlapped with known pre-miRNA will be taken as a re-identified pre-miRNA.

### 3.3. Deep Sequencing Data Analysis and New miRNAs Identification

The small RNA sequencing data from chimpanzee, gorilla, orangutan and rhesus macaque [28,29] has been re-analyzed to discover novel miRNAs and also been used to validate the predicted results by the above method. These deep sequencing data were processed with miRDeep2 [30] by using the parameters described in the previous reference [28]. Comparing to the previous study [28], we added human and mouse mature miRNAs as the known mature sequences when running miRDeep2. Moreover, another difference is that human miRNA and pre-miRNA data has been updated in miRBase 19.

### 3.4. Target Gene Ontology Analysis and Transcription Factors and Transcription Factor Binding Sites Exploration

Targets of those validated miRNAs were predicted using TargetScan 5.2 [31] and PITA [32] respectively. The intersection of TargetScan and PITA targets was used to do gene ontology annotation analysis. Enrichment of GO terms in miRNA predicted targets was performed using the GOstats Bioconductor package [41].

The transcription factors and transcription factor binding sites of those miRNAs which targets involve in the nervous system have been predicted by using the nucleosome-DNA interaction model [36,37] and vertebrate position frequency matrixs (PFMs) from JASPAR [38]. The potential regulatory sequence is extracted from the start site of the intronic of the host coding-gene in which one miRNA locates to the start site of the pre-miRNA if the miRNA is an intronic miRNA, while if a miRNA is an intergenic miRNA or a protein-coding gene, the sequence will be extracted from the upstream 2000 bp of the start site of the pre-miRNA or the protein-coding gene. Moreover, the upstream sequence has been separated into multiple sequences by uncertainty “Ns” which are unknown bases in the upstream sequence before prediction.

## 4. Conclusions

In this report, we first introduced a new approach to identify the pre-miRNAs in a target organism. It takes advantage of the synteny information between a reference genome and a target genome to find the orthologous miRNAs, especially to identify orthologs of highly divergent miRNAs. With this method, we identified 929 potential pre-miRNAs in marmoset in which miRNAs have not yet been reported and we successfully found 721, 979, 650 and 639 new potential pre-miRNAs in chimpanzee, gorilla, orangutan and rhesus macaque, respectively. These data show that our approach can effectively identify pre-miRNAs by using the synteny information. Then 56, 25, 16 and 29 predicted pre-miRNAs have been validated by the small RNA sequencing data from the brain or liver tissues in chimpanzee, gorilla, orangutan and rhesus macaque, respectively. Moreover, we found some of the validated miRNAs may play roles in nervous system and we also found some of them could be regulated by some common TFs.

## Figures and Tables

**Table 1:** The number of pre-miRNAs predicted by our method and new pre-miRNAs identified from small RNA sequencing data. The potential pre-miRNAs were identified by MiPred program. ND: No data; UA: The small RNA sequencing data is unavailable.

Primates	miRBase 19 (Known miRNA Genes)	Candidate miRNA Genes		Supported by Small RNA Sequencing Data	New miRNA Genes Identified from Deep Sequencing Data

		Re-identified miRNA Genes	Novel miRNA Genes		
Human	1600	ND	ND	ND	ND
Chimpanzee	655	601	721	56	234
Gorilla	322	301	979	25	237
Orangutan	633	590	650	16	393
Rhesus macaque	535	480	639	29	230
Marmoset	0	0	929	UA	UA

**Table 2:** Some validated miRNAs and their host genes in chimpanzee and orangutan. The validated miRNAs which targets are potentially involved in nervous system are located in protein-coding genes. Moreover, the number that follows “intron_” in this table means the miRNA location in which intron of the corresponding protein-coding gene. These coordinates of protein-coding genes and miRNAs of chimpanzee were derived from panTro4 assembly instead of panTro3. Moreover, all the sequence information of chimpanzee in this study was derived from panTro3 assembly if not explained specially.

Chromosome	Start	End	Gene Symbol	Intron	Strand	Chromosome	Start	End	pre-miRNA	Strand
chr1	196871470	196873147	PHC2	intron_11	+	chr1	196873044	196873102	ppy-mir-3605	+
chr7	130301326	130318076	EXOC4	intron_3	+	chr7	130303121	130303184	ppy-mir-4419a	+
chr19	51404019	51404127	PTOV1	intron_2	+	chr19	51404069	51404128	ppy-mir-4706	+
chr20	13523168	13526970	POLR3F	intron_2	+	chr20	13524745	13524802	ppy-mir-3192	+
chr17_random	2435896	2459049	SKA2	intron_1	+	chr17_random	2439995	2440051	ppy-mir-301a	+
chr3	49052547	49062118	SPINK8	intron_2	−	chr3	49059002	49059061	ptr-mir-2115	−
chr4	176472868	176516173	GALNT7	intron_2	+	chr4	176492588	176492646	ptr-mir-548t	+
chr11	65715927	65719260	NDUFS8	intron_6	+	chr11	65716414	65716476	ptr-mir-4691	+
chr13	102152614	102687690	FGF14	intron_4	−	chr13	102247593	102247653	ptr-mir-2681	−
chr15	63169799	63174675	DENND4A	intron_21	−	chr15	63171059	63171120	ptr-mir-4511	−
chr21	26267629	26354246	DSCAM	intron_22	−	chr21	26290766	26290830	ptr-mir-4760	−
chr22	44543820	44616196	ATXN10	intron_9	+	chr22	44567304	44567367	ptr-mir-4762	+
